# Severe allo-immune antibody-associated peripheral and central nervous system diseases after allogeneic hematopoietic stem cell transplantation

**DOI:** 10.1038/s41598-021-87989-z

**Published:** 2021-04-19

**Authors:** Martin W. Hümmert, Michael Stadler, Lothar Hambach, Stefan Gingele, Martin Bredt, Mike P. Wattjes, Gudrun Göhring, Letizia Venturini, Nora Möhn, Martin Stangel, Corinna Trebst, Arnold Ganser, Florian Wegner, Thomas Skripuletz

**Affiliations:** 1 Department of Neurology, Hannover Medical School, Carl-Neuberg-Str. 1, 30625 Hannover, Germany; 2 Department of Clinical Neuroimmunology and Neurochemistry, Hannover Medical School, Hannover, Germany; 3 Department of Hematology, Hemostasis, Oncology and Stem Cell Transplantation, Hannover Medical School, Hannover, Germany; 4 Hannover Medical School, Institute for Pathology, Hannover, Germany; 5 Department of Diagnostic and Interventional Neuroradiology, Hannover Medical School, Hannover, Germany; 6 Department of Human Genetics, Hannover Medical School, Hannover, Germany

**Keywords:** Demyelinating diseases, Haematological cancer

## Abstract

Allogeneic hematopoietic stem cell transplantation (alloHSCT) is a curative treatment for hematologic malignancies. Acute and chronic graft-versus-host disease (GvHD) are the major immune-mediated complications after alloHSCT. However, there is controversy whether neurologic complications after alloHSCT might represent manifestations of GvHD. We report three patients who acquired distinct, severe immune-mediated peripheral or central nervous system diseases after alloHSCT without other, concomitant GvHD manifestations. One patient had been diagnosed with B-cell chronic lymphocytic leukemia and two patients with high risk myelodysplastic syndrome. Patient #1 presented as LGI1- and GAD-IgG positive immune-mediated encephalitis, patient #2 was diagnosed with MOG-IgG positive encephalomyelitis, and patient #3 had chronic inflammatory polyneuropathy associated with SSA(Ro)-IgG positive Sjögren’s syndrome. 100% donor chimerism was detectable in the peripheral blood in all three. The specific antibodies were undetectable in donors’ and patients’ blood before alloHSCT suggesting that the antibodies had arisen from the transplanted donor immune system. Early intensive immunotherapy led to improvement of clinical symptoms and stability of the neurological disease, however, at the cost of losing the graft-versus-malignancy effect in one patient. In conclusion, we provide evidence of isolated, severe allo-immune diseases of the peripheral and central nervous system as complications of alloHSCT (“neuro-GvHD”). Interdisciplinary surveillance and thorough diagnostic work-up are needed for early diagnosis and treatment of neuro-immunologic complications after alloHSCT to improve the otherwise poor outcome.

## Introduction

Allogeneic hematopoietic stem cell transplantation (alloHSCT) provides a curative option for many life-threatening diseases, especially hematologic malignancies. Neurologic complications have been shown to significantly increase the morbidity and mortality after alloHSCT^1^. A recently published systematic review with meta-analysis evaluating 57,972 patients from different study designs revealed a relevant incidence of neurologic complications after alloHSCT^2^. The most important triggers and risk factors for neurologic complications after alloHSCT are drug-, radiation- and metabolic-related toxicity, infections (especially due to immunodeficiency), cerebrovascular and immune-mediated events^3^. Acute and chronic graft-versus-host diseases (GvHD) are the major immune-mediated complications after alloHSCT. Other immune-mediated complications, such as thyroiditis, rheumatoid arthritis or systemic lupus erythematosus (SLE) can occur^4^. Immune-mediated neurologic complications after alloHSCT are rare and often associated with poor outcomes. In a retrospective study with 3305 patients, only twelve (0.4%) developed an immune-mediated neuropathy following alloHSCT^5^. Another large retrospective study with 1484 patients after alloHSCT detected 3 patients (0.2%) with acute demyelinating encephalomyelitis and three further patients (0.2%) acquiring an acute inflammatory neuropathy^6^. These low incidence rates are supported by the aforementioned meta-analysis with a pooled incidence of immune-mediated neurologic complications including GvHD of 0.6%^2^. While GvHD is caused by the transplanted allo-immune system, the mechanisms of other immune-mediated complications including those affecting the nervous system are controversial and may involve both allo- and auto-immune responses. For example, myasthenia gravis is a well characterized antibody-related neurological complication after alloHSCT that usually occurs in context of GvHD^7,8^. Early diagnosis and treatment of neuro-immunological complications after alloHSCT may be very challenging because symptoms overlap with typical post HSCT complications such as toxicity-related polyneuropathy or fatigue.

Here, we report three allotransplanted patients with distinct antibody-associated neurologic diseases having occurred in the absence of clinical signs of concomitant GvHD.

## Methods

### Patients and procedures

AlloHSCT was performed at the Department of Hematology, Hemostasis, Oncology and Stem Cell Transplantation, and neurologic complications were treated at the Department of Neurology, both at Hannover Medical School, Germany. The study was approved by the ethics committee of the Hannover Medical School, Germany (no. 1322-2012). All research was performed in accordance with the Declaration of Helsinki. All patients gave written informed consent for publication.

### Cerebrospinal fluid (CSF) and serum analytical procedures

CSF was obtained via lumbar puncture and immediately analyzed in the Neurochemistry Laboratory of the Department of Neurology as reported previously^9^.

For monospecific testing on the presence of human IgG antibodies to neuronal or glial cells antigens, recombinant cell-based assays were used (Euroimmun, Lübeck, Germany). Biochips with a mosaic of HEK293 cells transfected with several neuronal surface antigens, i.e. leucine-rich glioma-inactivated protein 1 (LGI1) and myelin oligodendrocyte glycoprotein (MOG), were co-incubated with diluted patients’ sera, followed by fluorescent secondary anti-human antibodies and assessment using fluorescence microscopy. Anti-glutamic acid decarboxylase (GAD) autoantibodies were detected using immunoblots with recombinant antigens (PNS-Blot, Ravo Diagnostika, Freiburg, Germany) for screening test and followed by indirect immunohistochemistry using commercially available cerebellum primate slides (INOVA Diagnostics, San Diego, California, USA) for verification test in accordance with the manufacturer´s instructions. Antibody titration was carried out by indirect immunofluorescence (LGI1- and MOG-IgG) or indirect immunohistochemistry (GAD-IgG) through increasing dilution of the patient sample. The starting dilution was 1:10 (LGI1- and MOG-IgG) or 1:100 (GAD-IgG) for serum and undiluted for CSF. The samples were rated by two independent investigators who were blinded for the clinical data. Extractable nuclear antigens, like anti-SSA(Ro)-antibodies, were detected by ELISA^10^.

### Nerve conduction studies and magnetic resonance imaging (MRI)

In accordance with the recommendations of the International Federation of Clinical Neurophysiology each standardized electrodiagnostic examination included at least one median, ulnar, peroneal, tibial, and sural nerve conduction using superficial stimulators and recording electrodes^11,12^.

Patients with suspected central nervous system (CNS) involvement underwent a multisequence brain (patient #1 and #2) and/or spinal (patient #2) MRI protocol obtained on a 3 T or 1.5 T whole body MR system. The brain MRI protocol included a 3D fluid-attenuated inversion recovery (FLAIR) with multiplanar reconstructions, axial T2-weigthed, axial diffusion-weighted imaging (DWI), susceptibility-weighted images (SWI), and contrast-enhanced T1-weighted images. The spinal cord protocol included sagittal short tau inversion recovery, T2-weighted and contrast-enhanced T1-weighted images, as well as axial T2-weighted images. All images were evaluated by an experienced neuroradiologist with special expertise in the field of inflammatory diseases of the CNS.

### Chimerism analysis

Chimerism analysis was performed on full blood and on isolated CD4^+^ and CD8^+^ T cells with short tandem repeat polymerase chain-reaction as previously described^13^. CD4^+^ and CD8^+^ T cells were isolated by magnetic separation using the MicroBeads Kit and MACS Column (Miltenyi Biotec, Bergisch Gladbach, Germany) according to the instructions of the manufacturer. The purity of the positive selected cell fraction was determined by FACS analysis, ranging between 91 and 97%. Genomic DNA was isolated using the QIAamp DNA Micro kit (Qiagen, Hilden, Germany).

### Ethics declaration

The study was approved by the ethics committee of the Hannover Medical School, Germany (no. 1322-2012). All research was performed in accordance with the Declaration of Helsinki.

### Consent to participate/consent to publication

All patients gave their consent to participate and written informed consent for publication.

## Results

### Case 1

**Figure 1:**
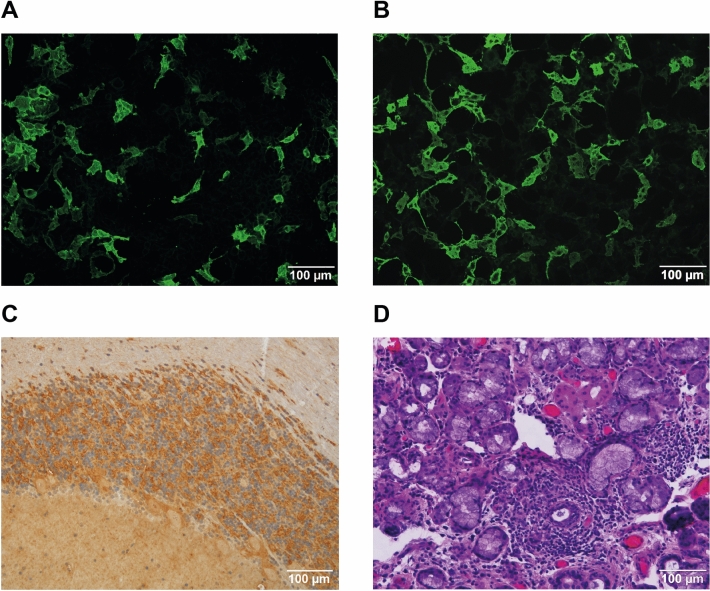
Immunofluorescent, immunohistochemical and histological findings. HEK 293 cells transfected with LGI-1 (**A**) or MOG (**B**) and incubated with patient´s sera (diluted 1:10) show positive cell immunolabeling after application of secondary fluorescent anti-human IgG. Anti-GAD-IgG immunoperoxidase staining in the primate cerebellum (**C**). Representative histological H&E-stained section with lymphocytic infiltration of the labial salivary gland with more than one focus [(**D**) sialadenitis grade IV according to Chisholm and Mason].

A 54-year-old male patient developed myoclonic jerks in the left shoulder 4 years after alloHSCT for B-cell chronic lymphocytic leukemia (B-CLL, for details see Table 1). Post-transplant complications had included mild and transient acute GvHD of the skin starting 4 months after alloHSCT. Due to incomplete donor chimerism he had received 3 donor lymphocyte infusions (DLIs) on days 174, 210 and 314 leading to conversion to complete donor chimerism and durable hematological remission. The neurological examination revealed intermittent brachial dystonic seizures on the left side of his body (see Supplementary Information 1). Neuropsychologic testing uncovered mild cognitive impairments in the areas of perceptual-motor skills, learning, and memory. The standard laboratory examination showed a known impaired kidney function and a slightly elevated creatine kinase. Brain MRI displayed no acute evidence of vascular or inflammatory/infectious pathology, particularly no imaging signs of encephalitis. Active neoplasia was ruled out by 18F-fluorodeoxyglucose PET/CT. Standard electroencephalography was normal. Detailed laboratory studies showed a decreased Vitamin D level (10.4 ng/ml, reference range: 20–50 ng/ml). Cerebrospinal fluid (CSF) analysis demonstrated a marginal pleocytosis (5 cells/µl, reference range < 5/µl) with detection of activated lymphocytes. CNS malignancy was ruled out by CSF cytology and FACS analysis. Moreover, a mild blood-CSF-barrier dysfunction (Q-albumin 9.9, protein 677 mg/l) and marginally positive oligoclonal bands (type 2a) without intrathecal immunoglobulin synthesis in Reiber graphs were found. Autoimmune encephalitis antibodies against the voltage-gated potassium channel associated LGI-1 protein with a serum titer of 1:40 and antineuronal antibodies against the GABA-synthesizing enzyme GAD with a serum titer of 1:1600 were detected (Fig. 1A,C), leading to the diagnosis of antibody-mediated immune encephalitis. After methylprednisolone (1 g/day for 5 days) and five cycles of immunoadsorption a therapy with the anti-CD20 antibody rituximab was established. In addition, an antiepileptic combination medication with valproic acid and levetiracetam was initiated. This therapy resulted in a significant reduction of motor and cognitive impairments, which remained stable in the long-term follow-up of 33 months under semi-annual rituximab infusion, allowing to discontinue valproic acid treatment.

**Table 1:** Patient characteristics.

Patient	Age (year)^a^/gender	Diagnosis	Conditioning	Donor/gender	CMVR/D	Stem cell source	GvHD prophylaxis	Additional cell therapy	GvHD	Complications (other than neurological)	Neuro-immunological disease
#1	50/male	B-CLL, complex karyotype	Flu/(Bu)/Cy	MMUD (HLA-B)/male	–/–	PBSC	ATG/CsA/MTX	1. DLI, d174 2. DLI, d210 3. DLI, d314	aGvHD I° (skin), d115	None	LGI1-IgG^+^- and GAD-IgG^+^ encephalitis, d1485
#2	64/female	MDS/MPN-u, JAK2^wt^, CBL^mut.^; DIPSS + high risk	Flu/Bu	MUD/female	–/–	PBSC	ATG/CsA/MTX	SC-boost, d159	aGvHD I° (skin), d117	Poor graft function zoster ophthalmi-cus with ceratitis dendritica, d187 → acyclovir	MOG-IgG^+^ encephalo-myelitis, d201
#3	62/female	MDS-EB-2, DNMT3A^mut.^, SF3B1^mut.^, IPSS intermed.2, IPSS-r high risk	FLAMSA/Bu	MUD/female	–/–	PBSC	ATG/CsA/MMF	None	aGvHD III° (skin), d31 aGvHD II° (gut), d49	aHUS, d82 → plasmapheresis → eculizumab	SSA(Ro)-IgG^+^ chronic inflammatory polyneuropathy, d343

*B-CLL* B-cell chronic lymphocytic leukemia, *MDS* myelodysplastic syndrome, *MPN* myeloproliferative neoplasm, *DIPSS+* Dynamic International Prognostic Scoring System plus, *Flu* fludarabin, *Bu* busulfan, *Cy* cyclophosphamide, *FLAMSA* fludarabin + amsacrine + high-dose cytarabine + busulfan + cyclophosphamide, *MUD* matched unrelated donor, *MMUD* mismatched unrelated donor, *R/D *recipient/donor, *PBSC* peripheral blood stem cells, *ATG* anti thymocyte globuline, *CsA* cyclosporine A, *MTX* methotrexate, *MMF* mycophenolate mofetil, *DLI* donor lymphocyte infusion, *d* day since alloHSCT, *SC-boost* stem cell boost, *aGvHD* acute graft versus host disease, *PNP* polyneuropathy, *aHUS* atypical hemolytic uremic syndrome

^a^At diagnosis of hematologic malignancy.

### Case 2

**Figure 2:**
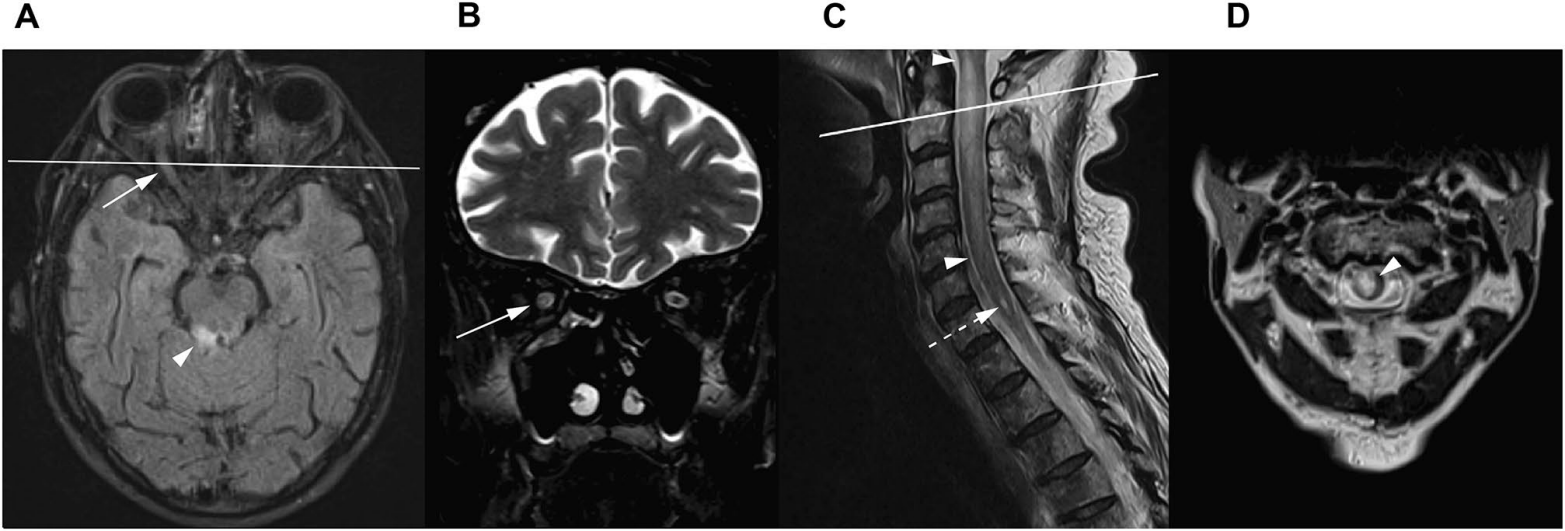
Pathological MRI findings. Axial FLAIR shows periependymal lesions involving the upper section of the pons (arrowhead, **A**) and upper cerebellar peduncle. Coronal fat-suppressed T2-weighted image shows hyperintense signal of the optic nerve, that is also visible on the axial FLAIR image (arrows, **A** and **B**). Sagittal T2-weighted spinal MRI disclosed a longitudinally extensive spinal cord lesion from C1-C5 (arrowheads indicate start and end of the lesion, **C** and **D**) and one lateral lesion at C7 (dashed arrow, **C**). The lines in the first axial and sagittal image indicate the localization of the respective coronar or axial layer.

A 65-year-old female patient was referred to our neurological department due to progressive left-sided choreatiform movement disorder, hemiparesis on the right, and visual loss on both eyes starting one month earlier. Onset of neurological symptoms was on day 201 after alloHSCT for high risk myelodysplastic/myeloproliferative syndrome. Post-transplant complications had included mild and transient acute GvHD of the skin at four months and delayed engraftment failure which was successfully treated by a T-cell-depleted stem cell boost five months after alloHSCT (for details see Table 1). Presentation at the neurological department was 1 month after treatment of zoster ophthalmicus with intravenous acyclovir for three weeks which entirely cured the skin lesions. Central nervous system zoster reactivation or other infections (CMV, HSV, EBV, borreliosis, and lues) as a possible reason for the neurological symptoms could be excluded by unremarkable cerebrospinal fluid analysis. Brain MRI revealed long-segment increased T2-weighted signal of the optic nerve in the middle portion and infratentorial periependymal lesions (Fig. 2A,B) both with contrast enhancement. Spinal cord MRI demonstrated a high signal T2-lesion involving five vertebral segments suggestive of extensive cervical myelitis (Fig. 2C,D) and one additional lower cervical lateral lesion (Fig. 2C). Comprehensive autoimmune laboratory testing resulted in positive MOG-IgG with a serum titer of 1:30 (Fig. 1B). Diagnosis of MOG-IgG associated encephalomyelitis was made^14^, and the patient was treated with methylprednisolone (500 mg/day for 5 days) followed by five cycles of immunoadsorption, tapered oral steroids, and rituximab every six months. Motoric complaints remitted completely, the visual acuity recovered successively, and no new neurologic deterioration occurred during 31 months of follow-up under regularly administered rituximab treatment. However, after the fifth course of rituximab, full donor chimerism was suddenly and completely lost, and the patient was again diagnosed with myelodysplastic/myeloproliferative syndrome, albeit in an earlier stage. The regular neurological treatment was therefore changed to high-dose immunoglobulins.

### Case 3

A 63-year-old female patient was admitted to our hospital because of rapidly progressive leg accentuated atrophic tetraparesis and paresthesia almost one year after alloHSCT for high risk myelodysplastic syndrome (for details see Table 1). Post-transplant complications had included transient neuropathic complaints by day 12 which recovered spontaneously, severe acute GvHD of the skin by day 31 and moderate GvHD of the gut by day 49, both transient. An atypical hemolytic uremic syndrome was diagnosed 3 months after alloHSCT and successfully treated with plasmapheresis and eculizumab. A simultaneously observed tetraparesis with electrophysiological identification of an axonal polyneuropathy was interpreted as critical illness polyneuropathy. Almost 1 year after alloHSCT progressive leg weakness with loss of walking ability and increasing numbness of all limbs over two months led to a detailed neurological examination. Nerve conduction studies revealed a severe axonal polyneuropathy with a significant deterioration compared to the previous measurement. Because of subjective and objective xerophthalmia and positive SSA(Ro)-autoantibodies a minor salivary gland biopsy was performed and uncovered a sialadenitis with more than one focus per 4 mm^2^ (Fig. 1D), but no fibrotic areas as seen in chronic GvHD-related dry eye disease^15^. Hence, chronic inflammatory polyneuropathy associated with Sjögren’s syndrome was diagnosed^12,16^. Since then, with the exception of a single interruption due to a severe infection, she received monthly intravenous immunoglobulin treatment, whereby clinical symptoms and electrophysiological measurements improved. The polyneuropathy was stable at last follow-up, 29 months after initiation of immunoglobulin treatment.

At onset of the neurological complaints, all patients had 100% donor chimerism for peripheral blood mononuclear cells (PBMCs), CD19^+^ B cells, CD4^+^ and CD8^+^ T cells, except for patient 1, who had 95% chimerism for CD19^+^ B cells (Table 2).

**Table 2:** Donor chimerism during neuro-immunologic disease.

Patient	Day after alloHSCT	% chimerism			
		PBMCs	CD4^+^ T cells	CD8^+^ T cells	CD19^+^ B cells
#1	d1519	100	100	100	95
#2	d241	100	100	100	100
#3	d440	100	100	100	100

LGI-1-, GAD-, MOG- and SSA(Ro)-IgG were neither detectable in the respective patient’s serum before alloHSCT nor in the respective donor’s serum.

## Discussion

We report three allotransplanted patients with severe, distinct antibody-associated neurologic diseases starting on days 201, 343 and 1485 after alloHSCT. Due to the stigmatizing movement disturbances and the associated high psychological strain, the diagnosis of neuro-immunologic diseases in patients #1 and #2 was barely delayed. With a less obvious symptom constellation (increasing paresis and numbness in patient #3), early diagnosis can be hampered or mimicked by the large variety of complications associated with alloHSCT, such as infections or drug-induced toxicities. The three patient examples outlined are the only isolated neuro-immunological complications we have encountered in all of our 1516 consecutive alloHSCT patients since 1995, amounting to an incidence of 0.2%. The rarity of neuro-immunological complications after alloHSCT underscore the need for prospective neurological surveillance of all transplanted patients with early interdisciplinary case discussion and detailed clinical and paraclinical assessment to avoid misdiagnosis and prevent long-term sequelae. This might also help to identify new risk factors for the complex field of neurologic complications after alloHSCT in order to prevent or counteract them.

All neuro-immunologic diseases were antibody-associated, and intensive immunotherapy including steroids, antibody-eliminating immunoadsorption, intravenous immunoglobulins and/or B cell depletion by rituximab led to improvement and disease stability in all three patients. Nevertheless, the pathophysiological mechanisms of the outlined neuro-immunologic diseases remain a matter of debate.

Considering the follow-up of 29–33 months without recurrence of malignancy after onset of immune-mediated neurologic disease, there is no evidence of paraneoplastic phenomena so far. Moreover, the detected antibodies are not known in a paraneoplastic context.

In patient #3, a toxic drug-induced neuropathy could be suspected due to the temporal correlation with the occurrence of the first neuropathic complaints; however, the rapidly progressive course after termination of the neurotoxic medication, the association with the diagnosis of Sjögren's syndrome and the stabilizing effect of immunoglobulins with transient worsening after an intravenous immunoglobulin-therapy pause, after which the clinical condition could only be stabilized by resuming intravenous immunoglobulin treatment, suggest an inflammatory genesis.

The delayed neuro-immune complication in patients #1 (4 years) and #3 (1 year) might be due to thymic damage with altered central tolerance mechanisms^4,17^.

Interestingly, all three patients were transplanted in a CMV^−/−^ constellation, i.e. with negative CMV serology in both recipient and donor, which only accounts for around 7% of all alloHSCT^18^. As previously shown, T cells regenerate with considerable delay after alloHSCT in the absence of CMV-specific cytotoxic T cells^19^. The disease association with antibodies and the treatment response to B cell-depleting therapies in our patients suggests a B cell driven pathology. It may be speculated whether a predisposition of low T cell levels may favor an excessive pathological activation of B cells with the development of antibody-mediated disease.

To our knowledge, only two other antibody associated cases with encephalitis after HSCT have been described in the literature to date^20,21^. Autoimmune encephalitides associated with antibodies against neuronal cell-surface proteins are relatively novel clinical entities. Since 2007, new antibodies are identified every year^22^. It may be hypothesized that some devastating encephalopathies in patients after alloHSCT without evidence of an infectious agent might be caused by currently unknown antibody-mediated diseases. Therefore, in cases of unexplained encephalopathies, a comprehensive antibody testing should be performed in an experienced laboratory whenever possible, as lack of or delayed immunotherapy may lead to severe sequelae or even death^23^.

All three patients described in this report had previously suffered from transient acute GvHD and accordingly showed complete donor chimerism (Table 2). However, while neurological manifestations developed, none of our patients suffered from concomitant acute or chronic GvHD as evidenced by clinical, laboratory, and functional assessments during regular follow-up visits. The temporal association (day 201) to alloHSCT in patient #2 might suggest a relation between transplantation and immune-mediated disease, possibly during immune reconstitution after stem cell boost, which was, however, T-cell depleted.

According to the consensus criteria of the National Institute of Health 2014, the diagnosis of any neurological manifestation of chronic GvHD requires the simultaneous existence of at least one typical diagnostic manifestation of chronic GvHD or at least one characteristic manifestation with a suitable additional test result^24^, which were altogether lacking in our three patients. CNS manifestations are omitted in these consensus criteria, but a number of cases combined with typical GvHD have been reported by others^20,25–28^. Furthermore, several reports of CNS manifestation without signs of systemic GvHD indicate a possible independent immune-mediated side effect after alloHSCT^29,30^, similar to our patients. Thus we are convinced, that the reported complications in our three patients represent isolated, allo-immune neurological disease (“neuro-GvHD”)^27^. In contrast, the possibility of an auto-immune-mediated disease driven by a small number of residual recipient plasma cells seems very unlikely. As previously published, a small percentage of plasma cells originating from the recipient can persist in the host organism and full plasma cell chimerism may not be reached in half of the patients after 1 year^31^. Nevertheless, none of our patients had shown the disease-related antibodies in the serum prior to alloHSCT, proposing that the complications were not caused by persisting recipient derived plasma cells surviving the conditioning before alloHSCT. Furthermore, disease-related antibodies were detectable in none of the donor sera before alloHSCT suggesting that the disorders were not caused by pre-existing antibodies transplanted from the donors. Only 1 of 3 patients had 5% residual recipient-derived B cells, while the 2 others displayed full chimerism in the B cell compartment (see Table 2). These data suggest that the disease-related antibodies had arisen in the patients only after alloHSCT and originated at least in 2 of 3 patients from donor-derived B cells.

Finally, the tilt and complete drop of full donor chimerism observed in patient #2 after rituximab suggests that the MOG-IgG associated encephalomyelitis had been due to a graft-versus-host mechanism which was lost during treatment—unfortunately together with its graft-versus-malignancy effect. This may also serve as a caveat and underscore the need to develop more specific, e.g. kinase inhibitor-based^32^, therapies against neuro-GvHD.

In conclusion, early, intermediate, and late onset of antibody-mediated severe immune-mediated diseases of the peripheral and central nervous system can occur after alloHSCT, which may represent neuro-GvHD with or without concomitant other GvHD manifestations. Beneficial long-term outcome based on early and intensive immunomodulatory therapy can be achieved in otherwise serious neurological autoimmune diseases that usually cause severe disability within a short period of time. Thus, a systematic, prospective approach with intense interdisciplinary exchange during follow-up of alloHSCT patients is needed to ensure early diagnosis of neurological complications. The scarce knowledge of the underlying pathophysiology on immune-mediated diseases after alloHSCT underlines the need for further clinical and experimental research in this field.

## Supplementary Information

Supplementary Video 1.

Supplementary Information.

## Data Availability

The datasets generated and/or analyzed during the current study are not publicly available but can be obtained from the corresponding author upon reasonable request.

## Abbreviations

alloHSCT: Allogeneic hematopoietic stem cell transplantation
[a]GvHD: [Acute] graft-versus-host disease
SLE: Systemic lupus erythematosus
CSF: Cerebrospinal fluid
LGI1: Leucine-rich glioma-inactivated protein 1
MOG: Myelin oligodendrocyte glycoprotein
GAD: Glutamic acid decarboxylase
MRI: Magnetic resonance imaging
CNS: Central nervous system
FLAIR: Fluid-attenuated inversion recovery
DWI: Diffusion-weighted imaging
SWI: Susceptibility-weighted images
B-CLL: B-cell chronic lymphocytic leukemia
DLIs: Donor lymphocyte infusions
MDS: Myelodysplastic syndrome
MPN: Myeloproliferative neoplasm
DIPSS+: Dynamic International Prognostic Scoring System plus
Flu: Fludarabine
Bu: Busulfan
Cy: Cyclophosphamide
FLAMSA: Fludarabin + amsacrine + high-dose cytarabine + busulfan + cyclophosphamide
MUD: Matched unrelated donor
MMUD: Mismatched unrelated donor
R/D: Recipient/donor
PBSC: Peripheral blood stem cells
ATG: Anti thymocyte globuline
CsA: Cyclosporine A
MTX: Methotrexate
MMF: Mycophenolate mofetil
DLI: Donor lymphocyte infusion
SC-boost: Stem cell boost
PNP: Polyneuropathy
aHUS: Atypical hemolytic uremic syndrome
PBMCs: Peripheral blood mononuclear cells
