# Supplementary material for: Severe allo-immune antibody-associated peripheral and central nervous system diseases after allogeneic hematopoietic stem cell transplantation
Source: Sci Rep. 2021 Apr 19;11:8527. doi: 10.1038/s41598-021-87989-z (PMC8055885; doi:10.1038/s41598-021-87989-z)
Supplement: Supplementary file 2 — Supplementary Information. [file 41598_2021_87989_MOESM2_ESM.docx]

**Supplementary information**

**Severe antibody-associated peripheral and central nervous system diseases after allogeneic hematopoietic stem cell transplantation**

Martin W. Hümmert1^*¶^, Michael Stadler^2*^, Lothar Hambach^2^, Stefan Gingele^1^, Martin Bredt^3^, Mike P. Wattjes^4^, Gudrun Göhring^5^, Letizia Venturini^2^, Nora Möhn^1^, Martin Stangel^1^, Corinna Trebst^1^, Arnold Ganser^2^, Florian Wegner^1*^, Thomas Skripuletz^1*^

^1^ Hannover Medical School, Dept. of Neurology and Dept. of Clinical Neuroimmunology and Neurochemistry, Hannover, Germany

^2^ Hannover Medical School, Dept. of Hematology, Hemostasis, Oncology and Stem Cell Transplantation, Hannover, Germany

^3^ Hannover Medical School, Institute for Pathology, Hannover, Germany

^4^ Hannover Medical School, Dept. of Diagnostic and Interventional Neuroradiology, Germany

^5^ Hannover Medical School, Dept. of Human Genetics, Germany

^*^ These authors contributed equally to this work.

**Additional file 1** (File format: .mp4)**.** Brachial dystonic seizures before and after first treatment course.
